# Supplementary material for: Aberration correction in long GRIN lens-based microendoscopes for extended field-of-view two-photon imaging in deep brain regions
Source: eLife. 2025 May 2;13:RP101420. doi: 10.7554/eLife.101420 (PMC12048154; doi:10.7554/eLife.101420)
Supplement: Supplementary file 1. — Parameters of Equation 1 (see Materials and Methods) for the simulated corrective lens designed to be applied to the GRIN rods of length 6.4 mm (top row) and 8.8 mm (bottom row). [file elife-101420-supp1.docx]

|  | ***c*** | ***k*** | ***α_1_*** | ***α_2_*** | ***α_3_*** | ***α_4_*** |
| --- | --- | --- | --- | --- | --- | --- |
| **6.4 mm-long GRIN rod** | 0.25 | 0 | -4.00 | 2.65 | 3.53 | -1.57 |
| **8.8 mm-long GRIN rod** | -0.36 | 0 | -0.94 | 23.40 | 349.33 | -720.06 |

**Supplementary File 1. Parameters of the polynomial function describing the aspherical surface of simulated corrective lenses.** Parameters of equation (*1*) (see Materials and Methods) for the simulated corrective lens designed to be applied to the GRIN rods of length 6.4 mm (top row) and 8.8 mm (bottom row).
