# Supplementary material for: Aberration correction in long GRIN lens-based microendoscopes for extended field-of-view two-photon imaging in deep brain regions
Source: eLife. 2025 May 2;13:RP101420. doi: 10.7554/eLife.101420 (PMC12048154; doi:10.7554/eLife.101420)
Supplement: Supplementary file 2. — Axial and lateral resolution of simulated microendoscopes were evaluated measuring the dimensions of simulated 2P PSF for each probe at different radial distances. x,z (Axial) and x,y (Lateral) intensity profiles of simulated PSFs were fitted with Gaussian curves and their FWHM was used to define the resolution, as done for experimental PSFs (see Materials and Methods). [file elife-101420-supp2.docx]

| **Microendoscope based on 6.4 mm-long GRIN rod** | | | | | |
| --- | --- | --- | --- | --- | --- |
| **Uncorrected** | | | **Corrected** | | |
| **Radial distance (µm)** | **Axial resolution (µm)** | **Lateral resolution (µm)** | **Radial distance (µm)** | **Axial resolution (µm)** | **Lateral resolution (µm)** |
| 0 | 12.7 | 1.6 | 0 | 7.3 | 0.7 |
| 89 | 39.4 | 2.7 | 65 | 7.3 | 0.7 |
| 127 | 29.5 | 14.4 | 93 | 9.0 | 1.2 |
| 155 | 29.9 | 19.0 | 115 | 11.2 | 1.2 |
| 179 | 31.6 | 23.4 | 135 | 13.7 | 1.3 |
| 200 | 24.5 | 24.9 | 155 | 19.9 | 1.6 |
| **Microendoscope based on 8.8 mm-long GRIN rod** | | | | | |
| **Uncorrected** | | | **Corrected** | | |
| **Radial distance (µm)** | **Axial resolution (µm)** | **Lateral resolution (µm)** | **Radial distance (µm)** | **Axial resolution (µm)** | **Lateral resolution (µm)** |
| 0 | 13.5 | 1.7 | 0 | 7.5 | 1.2 |
| 88 | 24.2 | 3.7 | 63 | 9.0 | 0.8 |
| 125 | 30.8 | 19.9 | 91 | 13.6 | 1.2 |
| 152 | 29.8 | 22.7 | 116 | 24.2 | 1.9 |

**Supplementary File 2. Spatial resolution of simulated uncorrected and corrected microendoscopes.** Axial and lateral resolution of simulated microendoscopes were evaluated measuring the dimensions of simulated 2P PSF for each probe at different radial distances. *x,z* (Axial) and *x,y* (Lateral) intensity profiles of simulated PSFs were fitted with Gaussian curves and their FWHM was used to define the resolution, as done for experimental PSFs (see Materials and Methods).
