# Supplementary material for: Aberration correction in long GRIN lens-based microendoscopes for extended field-of-view two-photon imaging in deep brain regions
Source: eLife. 2025 May 2;13:RP101420. doi: 10.7554/eLife.101420 (PMC12048154; doi:10.7554/eLife.101420)
Supplement: Supplementary file 3. — Coefficients of the quartic functions fitting the measurements performed on images acquired on the calibration ruler for uncorrected and corrected microendoscopes based on the 6.4 mm-long GRIN rod (left) and the 8.8 mm-long GRIN rod (right). The numbers in parenthesis indicate the 95% lower and upper confidence bounds (see Figure 3E and F). R-square values are indicated for each fit. [file elife-101420-supp3.docx]

| **Local magnification factor** | | | | | |
| --- | --- | --- | --- | --- | --- |
| **Microendoscope based on 6.4 mm-long GRIN rod**  *f(x) = ax^4^ + bx^2^ + c* | | | **Microendoscope based on 8.8 mm-long GRIN rod**  *f'(x) = a'x^4^ + b'x^2^ + c'* | | |
|  | **Uncorrected** | **Corrected** |  | **Uncorrected** | **Corrected** |
| *a* | -0.23∙10^-9^  (-1.12∙10^-9^, 0.66∙10^-9^) | -0.83∙10^-9^  (-1.09∙10^-9^,  -0.58∙10^-9^) | *a'* | 0.52 ∙10^-9^  (-1.52∙10^-9^, 2.56∙10^-9^) | -0.28∙10^-9^  (-0.56∙10^-9^, 0.0059∙10^-9^) |
| *b* | 0.15∙10^-5^  (-1.07∙10^-5^, 1.37∙10^-5^) | 0.10∙10^-4^  (0.036 ∙10^-4^, 0.17∙10^-4^) | *b'* | -0.27∙10^-5^  (-2.63∙10^-5^, 2.08∙10^-5^) | -0.18∙10^-4^  (-0.26 ∙10^-4^,  -1.07∙10^-4^) |
| *c* | 1.03  (1.00, 1.06) | 1.29  (1.25, 1.32) | *c'* | 1.04  (0.99, 1.09) | 1.36  (1.32, 1.40) |
| R-square | 0.12 | 0.93 | R-square | 0.13 | 0.98 |
| **Radial distance calibration** | | | | | |
| **Microendoscope based on 6.4 mm-long GRIN rod**  *g(x) = dx^4^ + ex^2^ + fx + h* | | | **Microendoscope based on 8.8 mm-long GRIN rod**  *g'(x) = d'x^4^ + e'x^2^ + f'x +h'* | | |
|  | **Uncorrected** | **Corrected** |  | **Uncorrected** | **Corrected** |
| *d* | -0.14∙10^-7^  (-0.31∙10^-7^, 0.33∙10^-7^) | -0.33∙10^-7^  (-0.40∙10^-7^,  -0.27∙10^-7^) | *d'* | 0.30∙10^-8^  (-2.28∙10^-8^, 2.88∙10^-8^) | -0.42∙10^-7^  (-0.46∙10^-7^,  -0.38∙10^-7^) |
| *e* | 0.22∙10^-3^  (-0.24∙10^-3^, 0.68∙10^-3^) | 1.03∙10^-3^  (0.68∙10^-3^, 1.37∙10^-3^) | *e'* | 0.45∙10^-4^  (-5.25∙10^-4^, 6.16∙10^-4^) | -0.16∙10^-4^  (-2.44∙10^-4^, 2.11∙10^-4^) |
| *f* | 1.03  (1.00, 1.06) | 1.24  (1.20, 1.27) | *f'* | 1.04  (1.00, 1.08) | 1.35  (1.32, 1.37) |
| *h* | -0.17  (-0.67, 0.33) | 0.13  (-0.63, 0.89) | *h'* | -0.18  (-0.70, 0.34) | -0.37  (-0.87, 0.14) |
| R-square | 1.00 | 1.00 | R-square | 1.00 | 1.00 |

**Supplementary File 3. Parameters used for the computation of the local pixel size and for distance calibration of images acquired with microendoscopes.** Coefficients of the quartic functions fitting the measurements performed on images acquired on the calibration ruler for uncorrected and corrected microendoscopes based on the 6.4 mm-long GRIN rod (left) and the 8.8 mm-long GRIN rod (right). The numbers in parenthesis indicate the 95% lower and upper confidence bounds (see Figure 3E, F). R-square values are indicated for each fit.
