# Supplementary material for: Aberration correction in long GRIN lens-based microendoscopes for extended field-of-view two-photon imaging in deep brain regions
Source: eLife. 2025 May 2;13:RP101420. doi: 10.7554/eLife.101420 (PMC12048154; doi:10.7554/eLife.101420)
Supplement: Supplementary file 4. — Coefficients of quartic functions fitting experimental PSF data (axial, top; lateral, bottom) are presented for uncorrected and corrected microendoscopes based on the 6.4 mm-long GRIN rod (left) and the 8.8 mm-long GRIN rod length (right). Parentheses indicate the 95% lower and upper confidence bounds (see Figure 3I and J). R-square values are indicated for each fit. [file elife-101420-supp4.docx]

| **Axial resolution** | | | | | |
| --- | --- | --- | --- | --- | --- |
| **Microendoscope based on 6.4 mm-long GRIN rod**  *f(x) = ax^4^ + bx^2^ + c* | | | **Microendoscope based on 8.8 mm-long GRIN rod**  *f'(x) = a'x^4^ + b'x^2^ + c'* | | |
|  | **Uncorrected** | **Corrected** |  | **Uncorrected** | **Corrected** |
| *a* | 0.41∙10^-6^  (-0.39∙10^-6^, 1.21∙10^-6^) | 0.20∙10^-7^  (-0.24∙10^-7^,  0.65 ∙10^-7^) | *a'* | 0.31∙10^-6^  (-1.28∙10^-6^, 1.91∙10^-6^) | -0.31∙10^-7^  (-0.63 ∙10^-7^, 0.014∙10^-7^) |
| *b* | 0.59∙10^-4^  (-43.64∙10^-4^, 44.83∙10^-4^) | -0.52∙10^-4^  (-8.53∙10^-4^, 7.48∙10^-4^) | *b'* | 0.19∙10^-2^  (-0.68∙10^-2^, 1.05∙10^-2^) | 0.12∙10^-2^  (0.053∙10^-2^, 0.18∙10^-2^) |
| *c* | 7.99  (4.20, 11.77) | 8.52  (6.03, 11.00) | *c'* | 7.43  (0.089, 14.78) | 7.04  (4.77, 9.30) |
| R-square | 1.00 | 0.72 | R-square | 1.00 | 0.93 |
| **Lateral resolution** | | | | | |
| **Microendoscope based on 6.4 mm-long GRIN rod**  *g(x) = dx^4^ + ex^2^ + f* | | | **Microendoscope based on 8.8 mm-long GRIN rod**  *g'(x) = d'x^4^ + e'x^2^ + f'* | | |
|  | **Uncorrected** | **Corrected** |  | **Uncorrected** | **Corrected** |
| *d* | 0.26∙10^-7^  (-2.83∙10^-7^, 3.35∙10^-7^) | 0.11∙10^-7^  (0.050∙10^-7^, 0.16∙10^-7^) | *d'* | -0.71∙10^-8^  (-22.84∙10^-8^, 21.42∙10^-8^) | 0.22∙10^-8^  (-0.047∙10^-8^, 0.50∙10^-8^) |
| *e* | 0.26∙10^-3^  (-1.45∙10^-3^, 1.97∙10^-3^) | -0.85∙10^-4^  (-1.87∙10^-4^, 0.17∙10^-4^) | *e'* | 0.13∙10^-3^  (-1.10∙10^-3^, 1.35∙10^-3^) | 0.59∙10^-4^  (0.031∙10^-4^, 1.15∙10^-4^) |
| *f* | 1.10  (-0.36, 2.56) | 1.37  (1.06, 1.69) | *f'* | 1.06  (0.0089, 2.10) | 1.12  (0.93, 1.31) |
| R-square | 0.99 | 0.95 | R-square | 0.94 | 0.98 |

**Supplementary File 4. Fitting parameters for PSF measurements of uncorrected and corrected microendoscopes.** Coefficients of quartic functions fitting experimental PSF data (axial, top; lateral, bottom) are presented for uncorrected and corrected microendoscopes based on the 6.4 mm-long GRIN rod (left) and the 8.8 mm-long GRIN rod length (right). Parentheses indicate the 95% lower and upper confidence bounds (see Figure 3I, J). R-square values are indicated for each fit.
