# Supplementary material for: Aberration correction in long GRIN lens-based microendoscopes for extended field-of-view two-photon imaging in deep brain regions
Source: eLife. 2025 May 2;13:RP101420. doi: 10.7554/eLife.101420 (PMC12048154; doi:10.7554/eLife.101420)
Supplement: Supplementary file 5. — Numerical values used to estimate the expected correlation between cell pairs in synthetic calcium t-series are indicated for each microendoscope type. The table displays the mean and SD of Pearson’s correlation between the activity traces of any possible ground truth source neuron pair obtained from n simulated FOVs and the expected cell pair correlation (mean Pearson’s correlation plus three SDs). These parameters were used for the analysis in Figure 6A and F and in Figure 6—figure supplement 2A and C. [file elife-101420-supp5.docx]

|  | **6.4 mm-long microendoscope**  (*n* = 13 FOVs) | | | **8.8 mm-long microendoscope**  (*n* = 15 FOVs) | | |
| --- | --- | --- | --- | --- | --- | --- |
|  | **Mean** | **Standard deviation** | **Expected pair correlation** | **Mean** | **Standard deviation** | **Expected pair correlation** |
| **Uncorrected** | 0.0393 | 0.0032 | 0.0488 | 0.0386 | 0.0031 | 0.0479 |
| **Corrected** | 0.0388 | 0.0026 | 0.0466 | 0.0389 | 0.0023 | 0.0458 |

**Supplementary File 5. Expected Pearson’s correlation of cell pair in synthetic calcium data.** Numerical values used to estimate the expected correlation between cell pairs in synthetic calcium t-series are indicated for each microendoscope type. The table displays the mean and SD of Pearson’s correlation between the activity traces of any possible ground truth source neuron pair obtained from *n* simulated FOVs and the expected cell pair correlation (mean Pearson’s correlation plus three SDs). These parameters were used for the analysis in Figure 6A, F and in Figure 6-figure supplement 2A, C.
