# Supplementary material for: Aberration correction in long GRIN lens-based microendoscopes for extended field-of-view two-photon imaging in deep brain regions
Source: eLife. 2025 May 2;13:RP101420. doi: 10.7554/eLife.101420 (PMC12048154; doi:10.7554/eLife.101420)
Supplement: Supplementary file 6. — The values of the slope of the linear fits are indicated ± s.e. for adjacent neurons with maximum centroid distance equal to 25 µm (top) or 30 µm (bottom). Results obtained with jGCaMP8f, jGCaMP7f, and with the merged dataset including both jGCaMP8f and jGCaMP7f are displayed. The number n of adjacent neuron pairs, the p value of the indicated statistical test for the normality of residuals, and the p value of the Wald test on the null hypothesis of slope = 0 are indicated for each condition. [file elife-101420-supp6.docx]

|  | **Adjacent neurons: distance between centroids ≤ 25 µm** | | |
| --- | --- | --- | --- |
|  | **jGCaMP8f (4 mice)** | **jGCaMP7f (2 mice)** | **Merge (6 mice)** |
| ***n*** | 168 | 27 | 195 |
| ***p* value (normality of residuals)** | D’Agostino-Pearson: 0.51 | Shapiro-Wilk: 0.74 | D’Agostino-Pearson: 0.29 |
| **slope (µm^-1^)** | -0.0006 ± 0.0004 | 0.002 ± 0.001 | -0.0006 ± 0.0004 |
| ***p* value (slope = 0)** | Wald test: 0.11 | Wald test: 0.18 | Wald test: 0.09 |
|  | **Adjacent neurons: distance between centroids ≤ 30 µm** | | |
|  | **jGCaMP8f (4 mice)** | **jGCaMP7f (2 mice)** | **Merge (6 mice)** |
| ***n*** | 249 | 39 | 288 |
| ***p* value (normality of residuals)** | D’Agostino-Pearson: 0.25 | Shapiro-Wilk: 0.62 | D’Agostino-Pearson: 0.42 |
| **slope (µm^-1^)** | -0.0005 ± 0.0003 | 0.001 ± 0.001 | -0.0005 ± 0.0003 |
| ***p* value (slope = 0)** | Wald test: 0.13 | Wald test: 0.18 | Wald test: 0.06 |

**Supplementary File 6. Linear regression analysis for pairwise correlation of adjacent neurons as a function of the radial distance of pair centroid for *in vivo* 2P imaging data.** The values of the slope of the linear fits are indicated ± s.e. for adjacent neurons with maximum centroid distance equal to 25 µm (top) or 30 µm (bottom). Results obtained with jGCaMP8f, jGCaMP7f, and with the merged dataset including both jGCaMP8f and jGCaMP7f are displayed. The number *n* of adjacent neuron pairs, the *p* value of the indicated statistical test for the normality of residuals, and the *p* value of the Wald test on the null hypothesis of slope = 0 are indicated for each condition.
